# Supplementary material for: Structural basis for peroxidase encapsulation inside the encapsulin from the Gram-negative pathogen Klebsiella pneumoniae
Source: Nat Commun. 2024 Mar 22;15:2558. doi: 10.1038/s41467-024-46880-x (PMC10960027; doi:10.1038/s41467-024-46880-x)
Supplement: Supplementary file 1 — Supplementary Information [file 41467_2024_46880_MOESM1_ESM.pdf]

Supplementary Information for

Structural basis for peroxidase encapsulation inside the encapsulin from the Gram-negative pathogen *Klebsiella pneumoniae*

Jesse A. Jones[1#], Michael P. Andreas[1#], and Tobias W. Giessen[1*]

[1]Department of Biological Chemistry, University of Michigan Medical School, Ann Arbor, MI, USA

[#]contributed equally

*correspondence: tgiessen@umich.edu

Table of Contents

Supplementary Fig. 1. Additional TEM micrographs of DyP-loaded KpEnc	2
Supplementary Fig. 2. Dynamic light scattering analysis of DyP-loaded KpEnc	3
Supplementary Fig. 3. Cryo-EM analysis of KpDyP_Enc	4
Supplementary Table 1. Cryo-EM data collection and model building statistics	5
Supplementary Fig. 4. KpEnc 5-fold pore characterization via MOLEonline	5
Supplementary Fig. 5. Structural analysis of the KpEnc 5-fold pore using MOLEonline	6
Supplementary Fig. 6. Dynamic light scattering analysis of free KpDyP	7
Supplementary Fig. 7. Analytical SEC and native PAGE analysis of free KpDyP	8
Supplementary Fig. 8. Cryo-EM analysis of free KpDyP	9
Supplementary Fig. 9. Electrostatic surface analysis of KpDyP	10
Supplementary Fig. 10. Cryo-EM analysis of SUMO-TP_Enc	11
Supplementary Fig. 11. Comparison of the TP binding site densities	12
Supplementary Fig. 12. Ionic and hydrogen bonding TP-shell interactions	13
Supplementary Fig. 13. Full SUMO-TP mutant SDS-PAGE gels	14
Supplementary Table 2. Protein sequences of constructs used in this study	15
Supplementary References	16

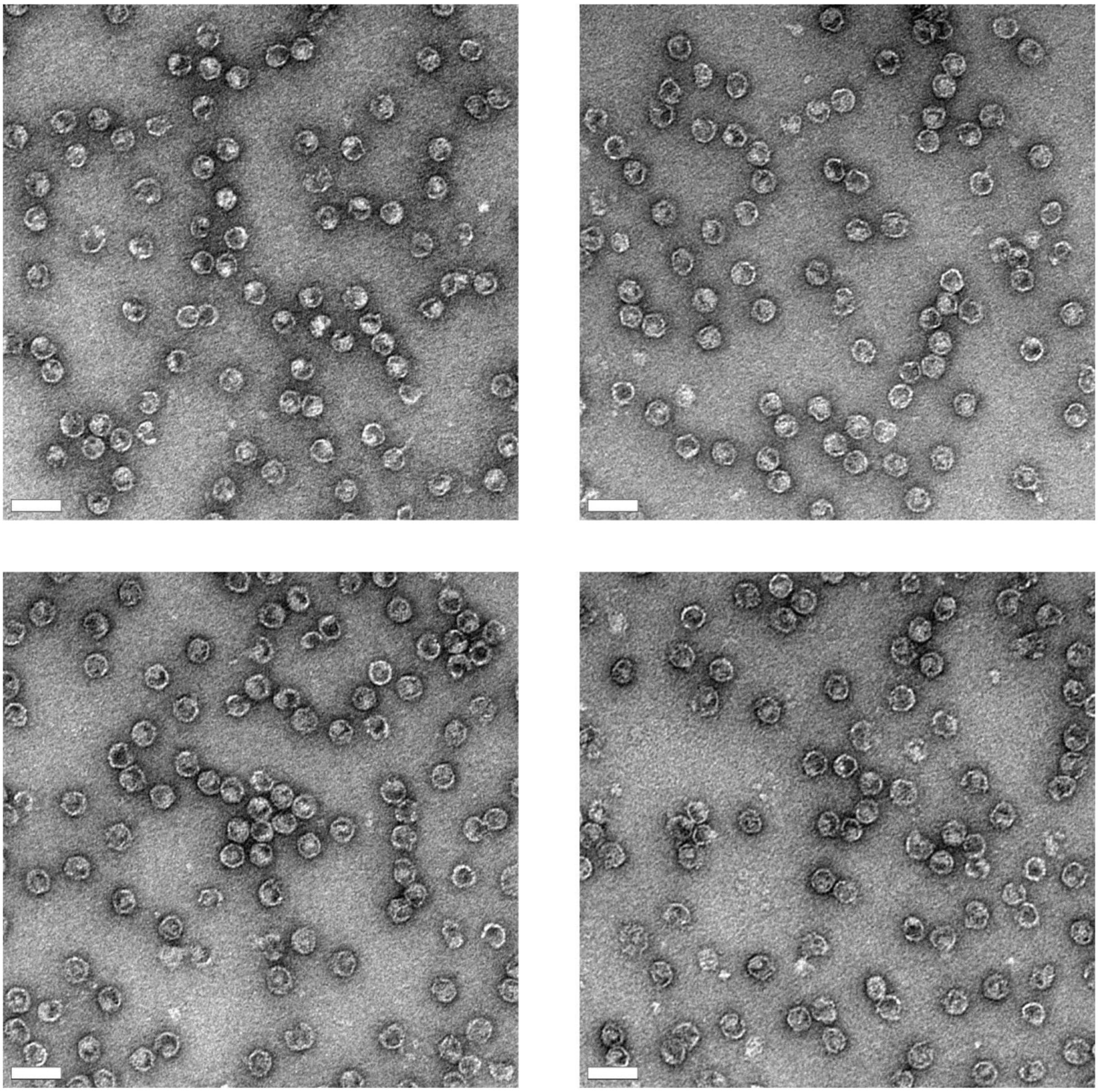

Supplementary Fig. 1 | Additional TEM micrographs of DyP-loaded KpEnc nanocompartments. TEM micrographs of separate KpDyP_Enc replicate samples. Scale bars (white): 50 nm.

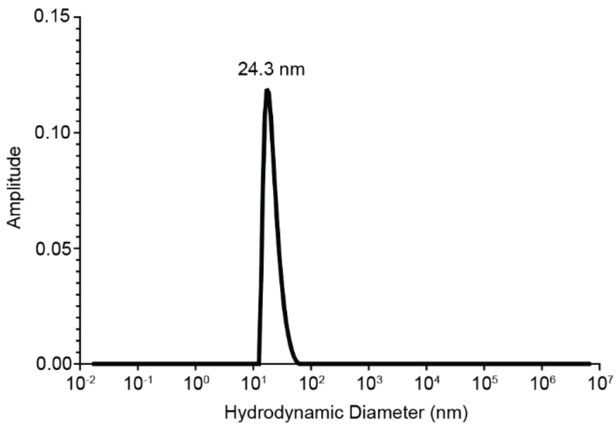

Supplementary Fig. 2 | Dynamic light scattering analysis of DyP-loaded KpEnc. DLS of DyP-containing KpEnc at pH 7.5 showing a Z-average diameter of 24.80 nm and a peak diameter of 24.30 nm.

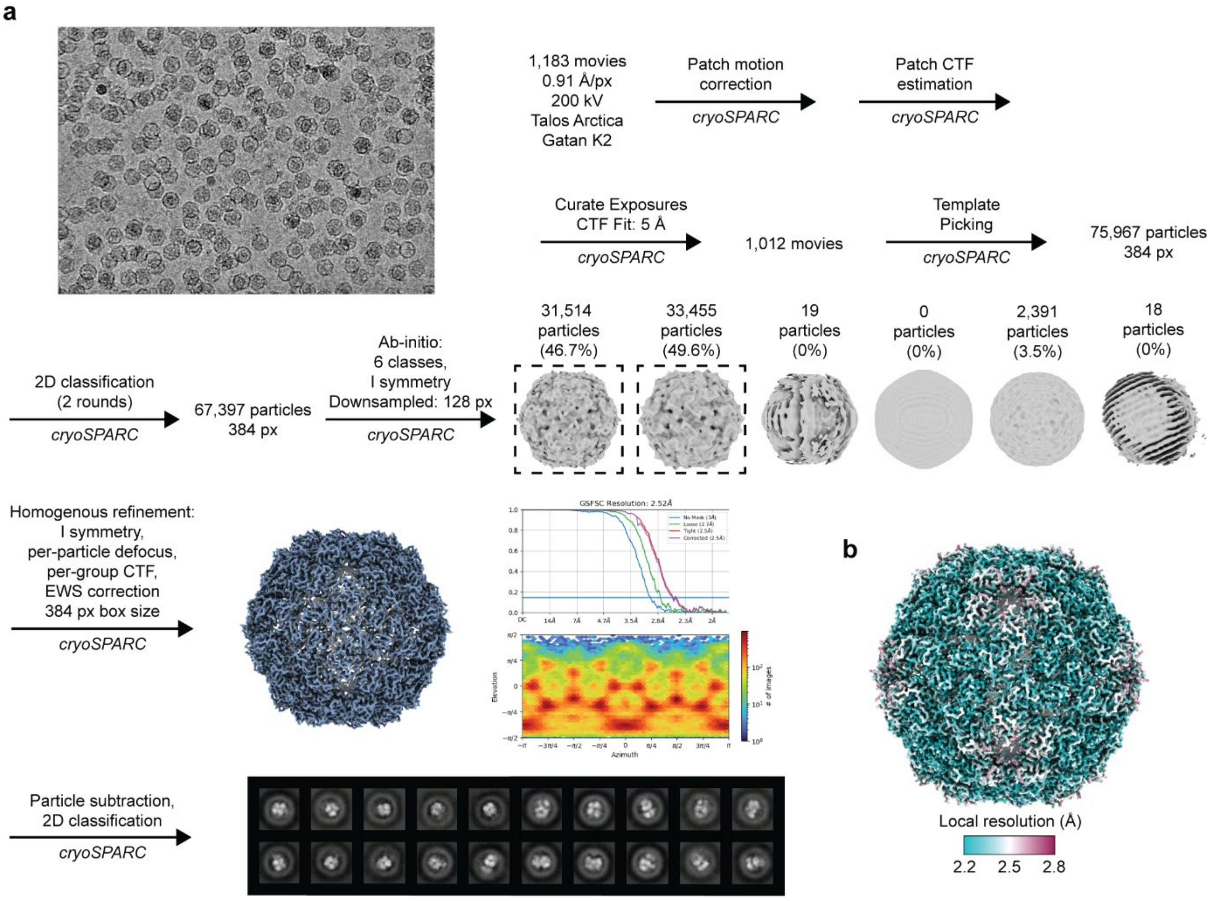

Supplementary Fig. 3 | Cryo-EM analysis of KpDyP_Enc.[1,2] **a,** Cryo-EM data processing workflow. **b,** Exterior view of the KpDyP_Enc shell colored by estimated local resolution.

Supplementary Table 1 | Cryo-EM data collection and model building statistics.[1,3-7]

	KpDyP_Enc (EMD-41905) (PDB 8U50)	SUMO-TP_Enc (EMD-41906) (PDB 8U51)	KpDyP (EMDB-41904) (PDB 8U4Z)
**Data collection and processing**			
Magnification	45,000x	45,000x	105,000x
Voltage (kV)	200	200	300
Electron exposure ($e^-/Å^2$)	41.67	39.58	50.02
Defocus range (μm)	-0.8 to -1.8	-0.8 to -1.8	-0.8 to -1.8
Pixel size (Å)	0.91	0.91	0.832
Symmetry imposed	I	I	D3
Initial particle images (no.)	75,967	115,682	2,603,020
Final particle images (no.)	64,969	101,111	431,317
Map resolution (Å)	2.52	2.41	2.39
FSC threshold	0.143	0.143	0.143
**Refinement**			
Initial model used (PDB code)	7BOJ	8U50	AlphaFill
Model resolution (Å)	2.8	2.7	2.7
FSC threshold	0.5	0.5	0.5
Map sharpening *B* factor ($Å^2$)	-95.2	-93.8	-107.7
Model composition			
Non-hydrogen atoms	2,026	2,093	2,466
Protein residues	267	277	313
Ligands	0	0	1
B factors ($Å^2$)			
Protein	43.67	32.62	45.69
Ligands	-	-	35.40
r.m.s. deviations			
Bond lengths (Å)	0.004	0.005	0.008
Bond angles (°)	0.979	1.020	1.226
Validation			
MolProbity score	1.37	1.36	1.39
Clashscore	6.73	4.34	5.14
Poor rotamers (%)	0.47	1.36	0
Ramachandran plot			
Favored (%)	98.49	97.80	97.43
Allowed (%)	1.51	2.20	2.57
Disallowed (%)	0	0	0

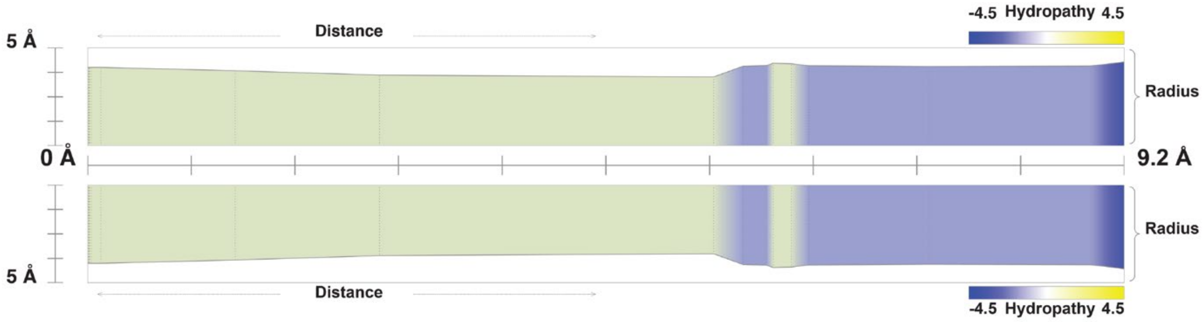

Supplementary Fig. 4 | KpEnc 5-fold pore characterization via MOLEonline.[8,9]

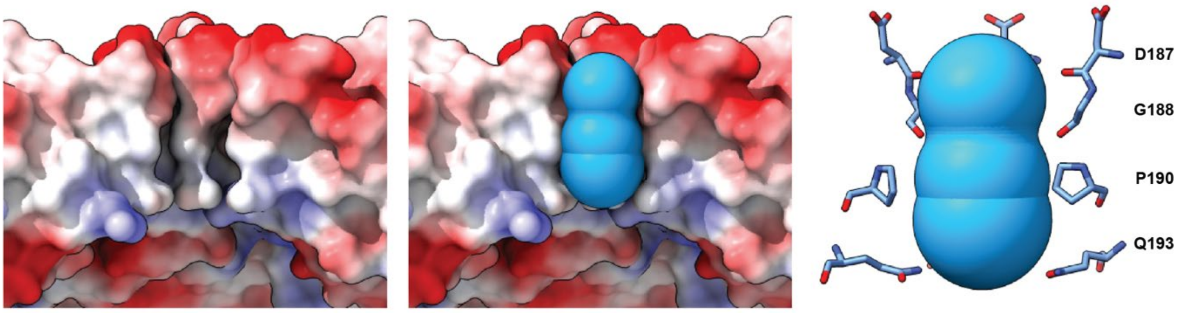

Supplementary Fig. 5 | Structural analysis of the KpEnc 5-fold pore using MOLEonline.[8,9] Electrostatic surface representation of the 5-fold pore. The residues of one subunit lining the 5-fold pore are shown.

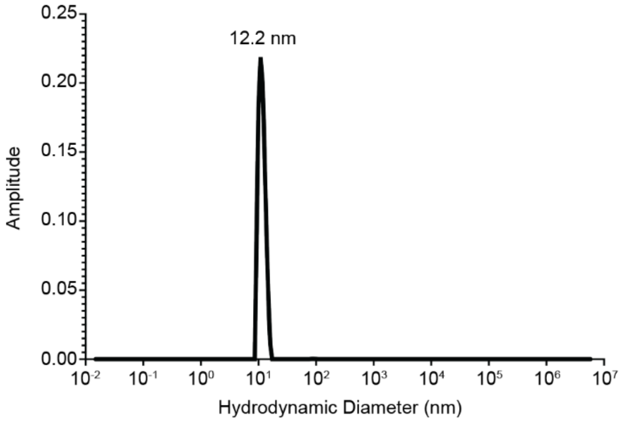

Supplementary Fig. 6 | Dynamic light scattering analysis of free KpDyP. DLS showing a Z-average diameter of 13 nm and a peak diameter of 12.21 nm for the purified free KpDyP at pH 7.5.

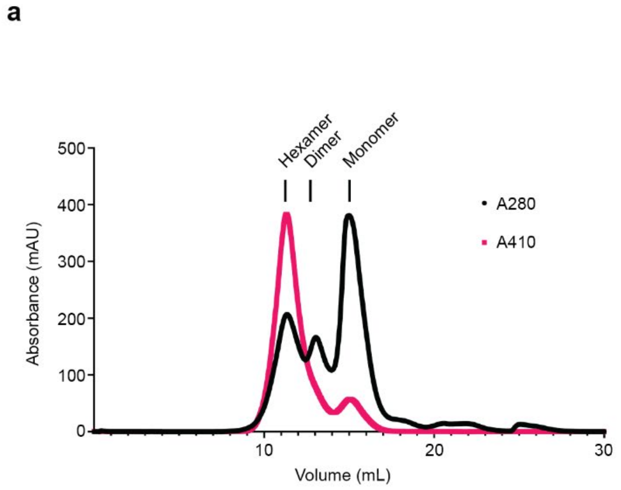

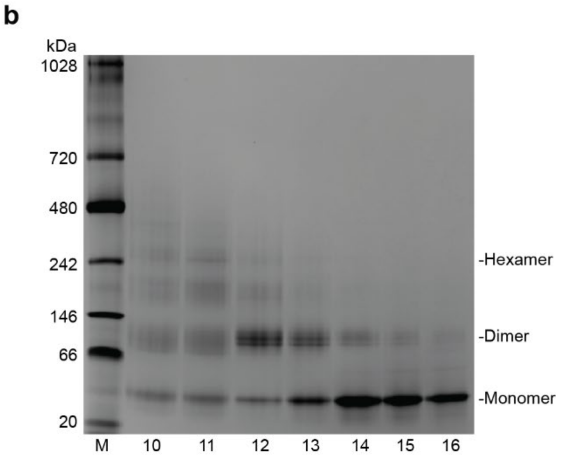

Supplementary Fig. 7 | Analytical SEC and native PAGE analysis of free KpDyP. **a,** Analytical SEC analysis of purified KpDyP using a Superdex 200 column. **b**, Native PAGE analysis of KpDyP-containing Superdex 200 fractions. Lane labels correspond to SEC mL. M, marker.

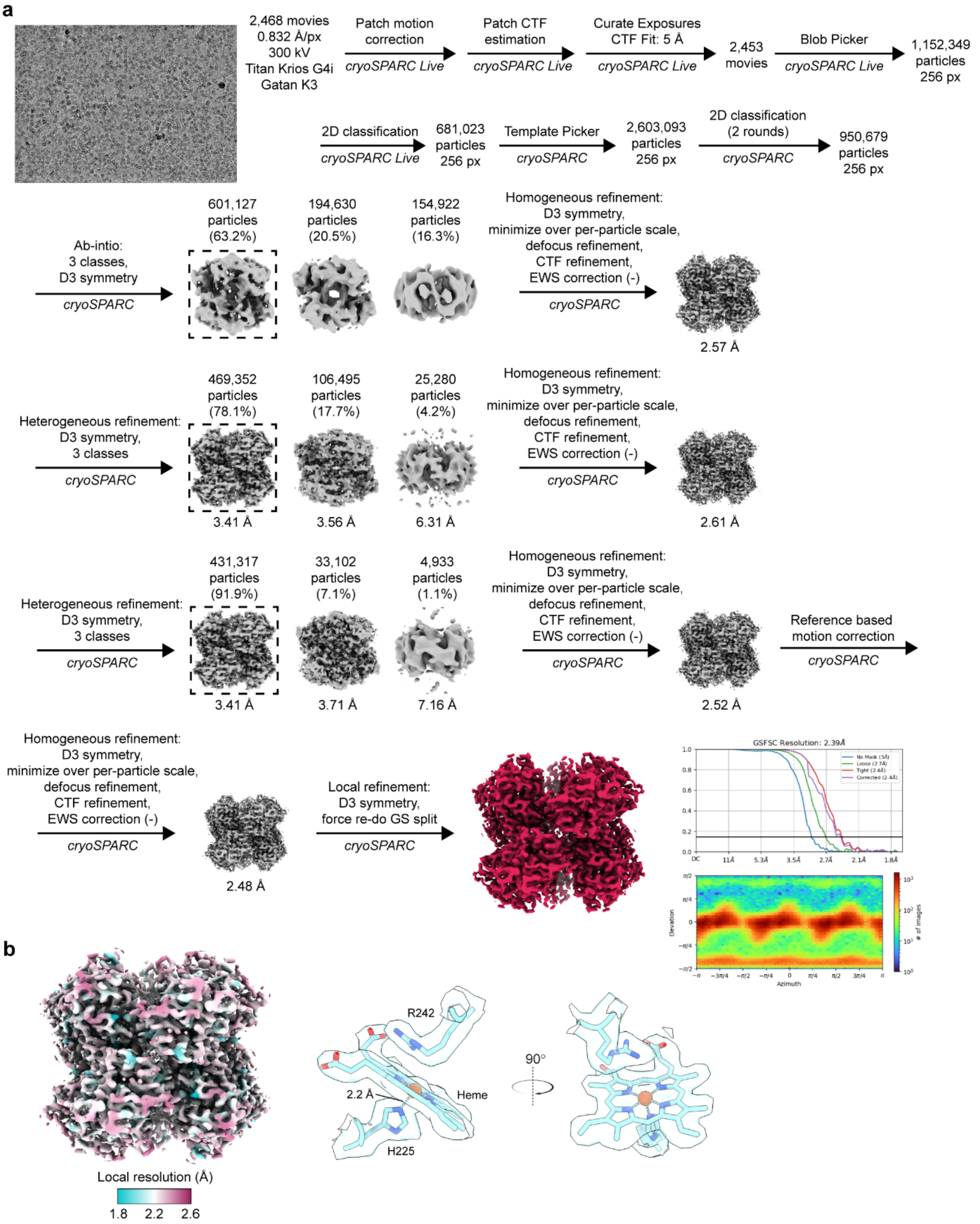

Supplementary Fig. 8 | Cryo-EM analysis of free KpDyP.[1,2] **a,** Cryo-EM data processing workflow. **b,** KpDyP hexamer colored by estimated local resolution (left) with magnified views of the heme density with atomic models for the heme moiety and His225 as well as Arg242 shown (middle and right).

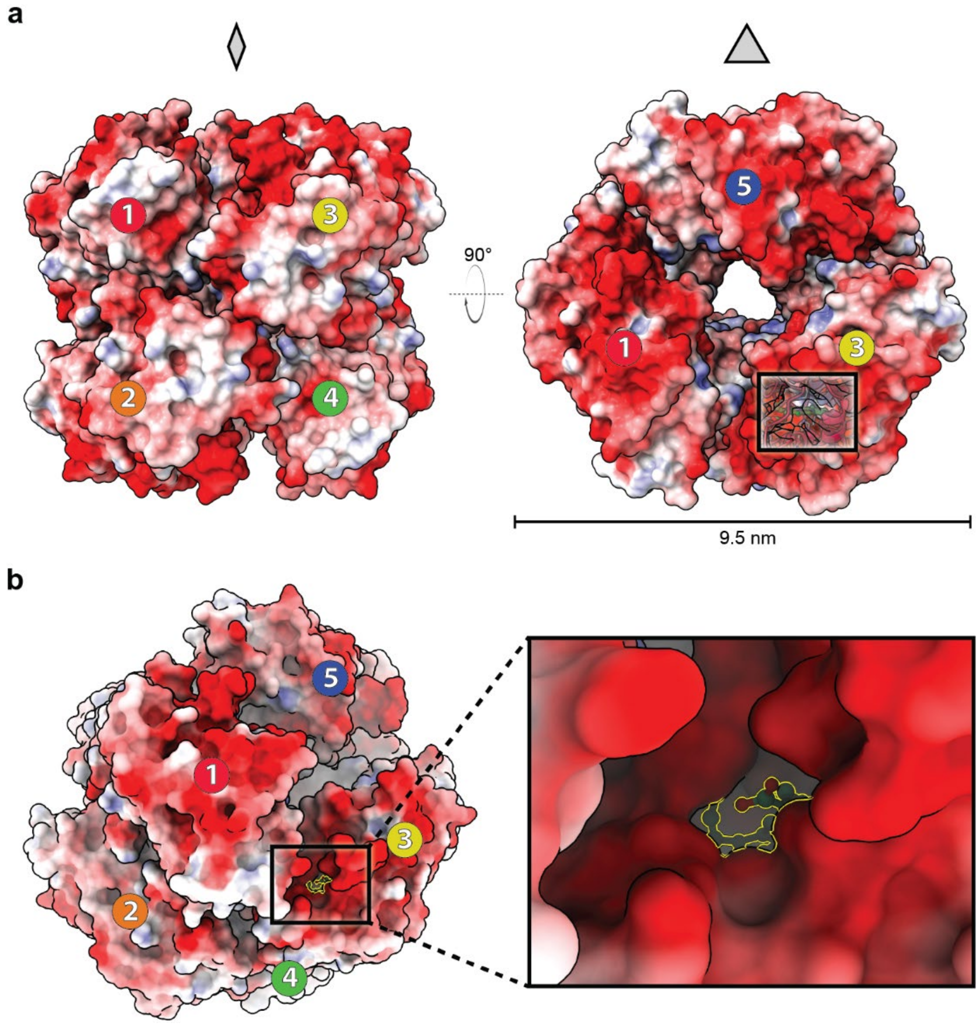

Supplementary Fig. 9 | Electrostatic surface and active site entrance analysis of KpDyP.[2] **a,** Electrostatic surface representation of the KpDyP hexamer along the two-fold symmetry axis (left) and three-fold symmetry axis (right) with individual subunits numbered and heme molecule highlighted (yellow) through transparent surface (right, black box). Theoretical isoelectric point (pI) was calculated to be 4.65 and the charge at pH 7.0 was calculated to be -21.70, with 54 negatively charged residues and 32 positively charged residues. **b,** Rotated model showing heme (highlighted yellow) as well as a zoomed in representation of a potential access channel to the active site (right).

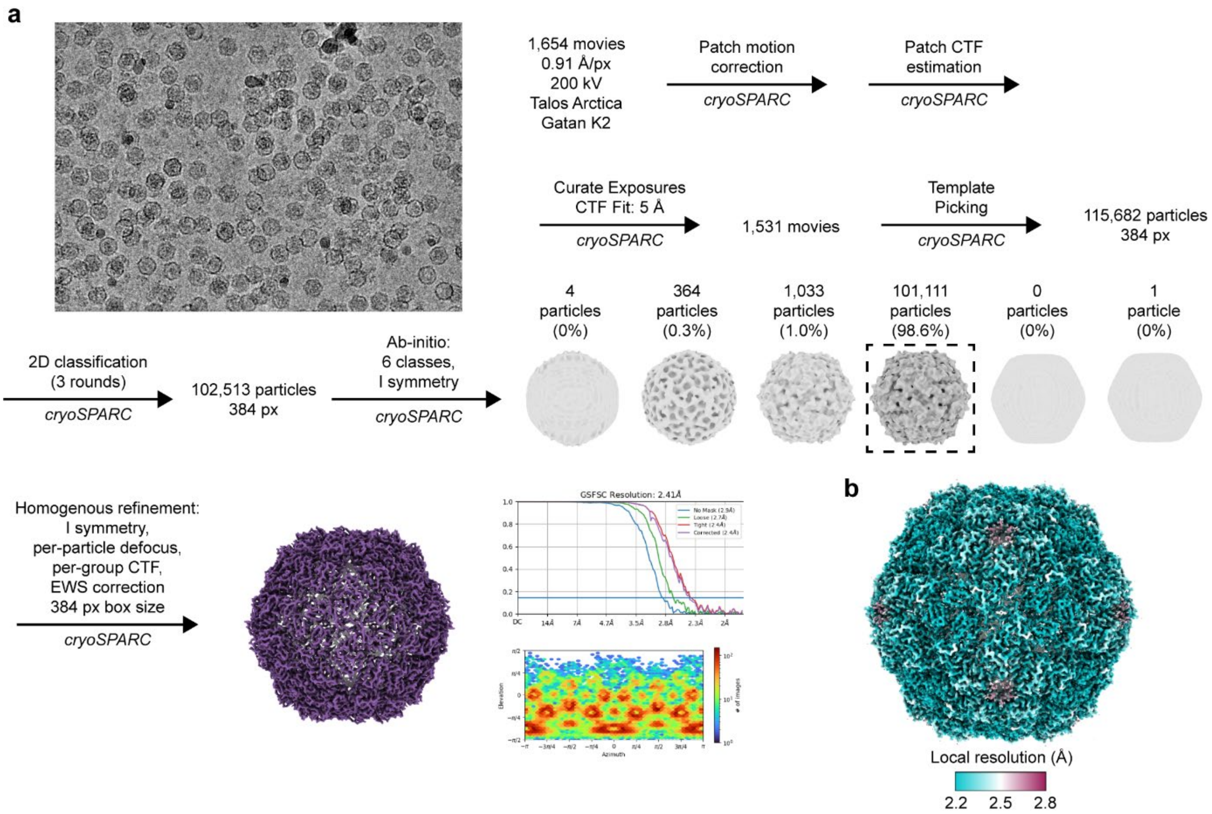

Supplementary Fig. 10 | Cryo-EM analysis of SUMO-TP_Enc.[1] **a,** Cryo-EM data processing workflow. **b,** Exterior view of the SUMO-TP_Enc shell colored by estimated local resolution.

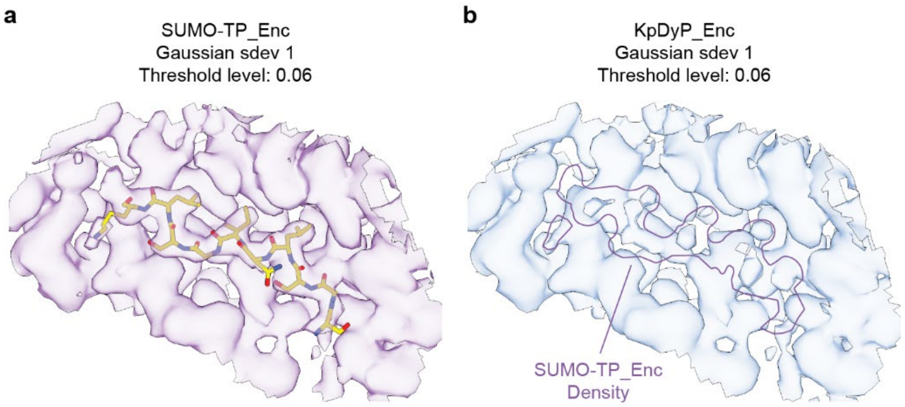

Supplementary Fig. 11 | Comparison of the TP binding site densities of KpDyP_Enc and SUMO-TP_Enc.[1,2] **a,** Cryo-EM density map of the KpEnc encapsulin interior surface and the bound TP from SUMO-TP_Enc (purple) with SGSLNIGSLK TP in stick representation (yellow) for map-to-model comparison. **b,** Cryo-EM density map of the TP binding site of KpDyP_Enc (blue). The outline of the TP density from SUMO-TP_Enc is shown and overlayed for comparison (purple).

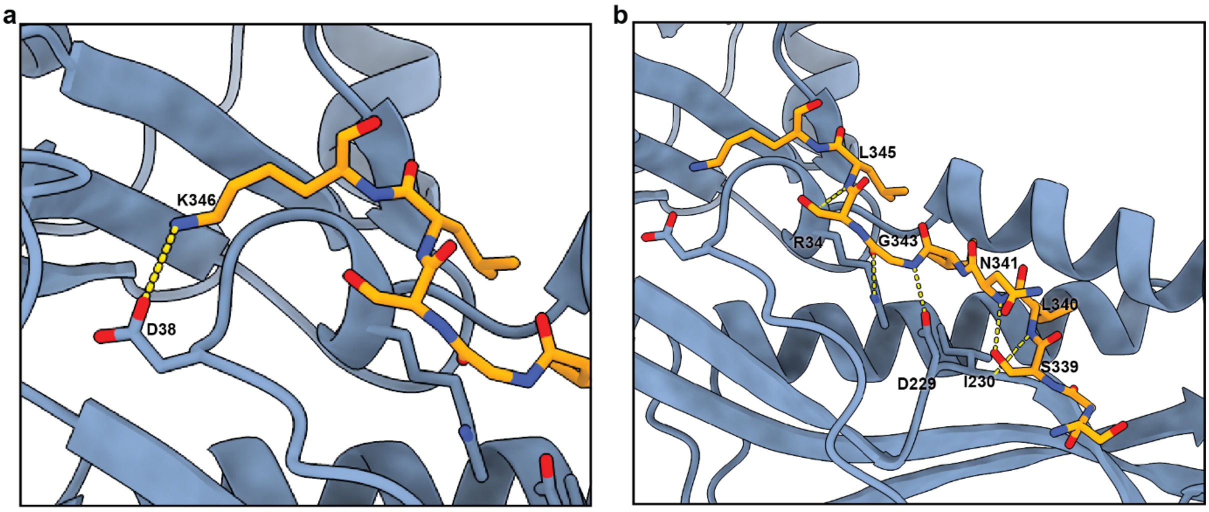

Supplementary Fig. 12 | Ionic and hydrogen bonding interactions mediating TP-shell binding. a, Salt bridge between TP residue Lys 346 and encapsulin shell protein residue Asp38. **b**, Inter- and intramolecular hydrogen bonds of TP residues.

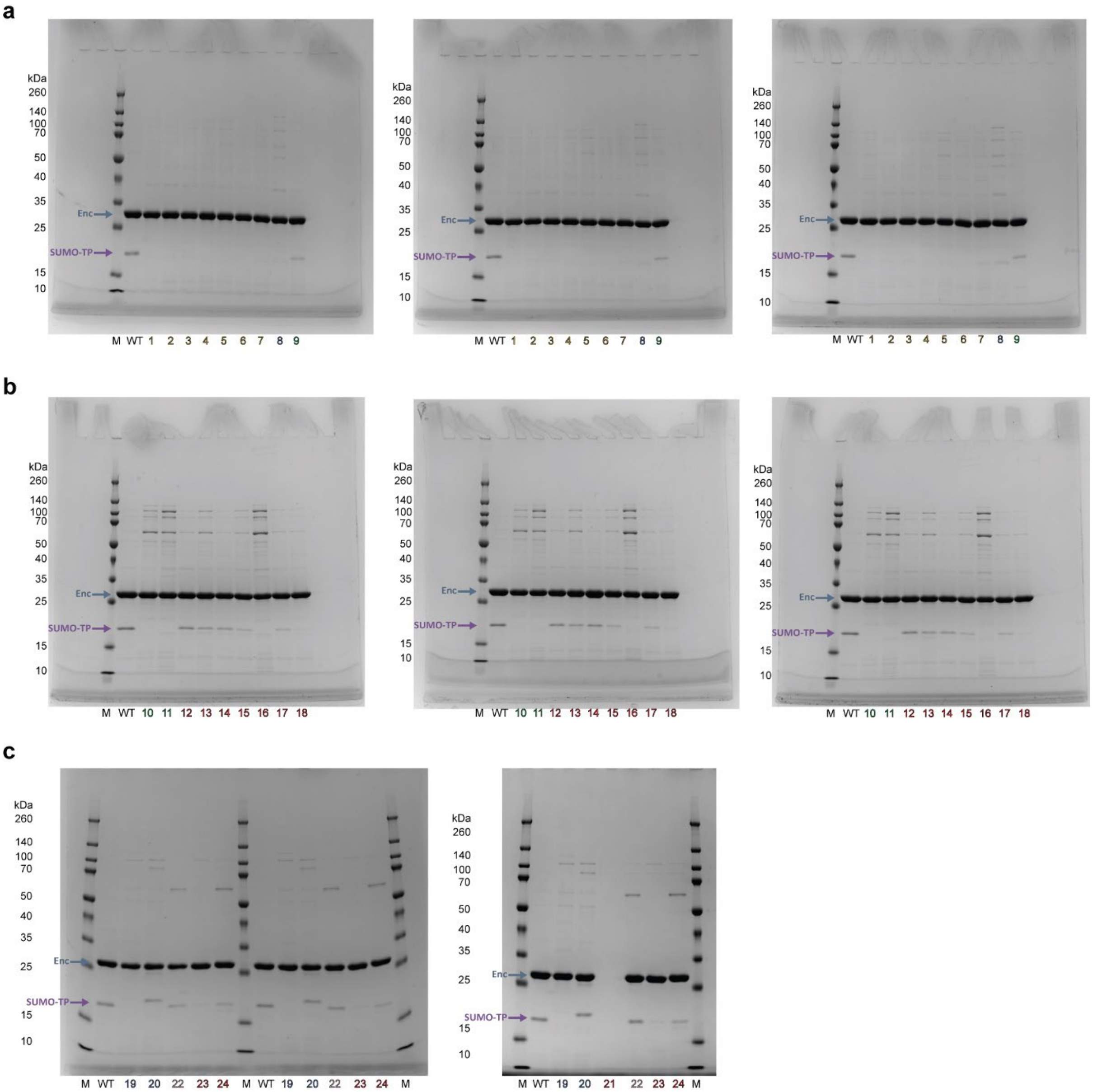

Supplementary Fig. 13 | Full SUMO-TP and KpEnc TP binding site mutants SDS-PAGE gels. a, Full triplicate SDS-PAGE gels illustrating cargo loading capacity of WT and respective mutants 1 through 9 with KpEnc (higher band, blue) and the co-purified encapsulated SUMO-TP mutant cargo (lower band, purple) where present. **b**, Same layout as above, but for mutants 10 through 18. **c**, Same layout as above, but for mutants 19 through 24.

Supplementary Table 2 | Protein sequences of constructs used in this study.

Construct	Protein sequence[1,2]
KpDyP_Enc	MACPISQSVSQPVDERLTRAAIFLVVTINPGKAAEVAVRTLCGTLSSLVR<br>GVGFRILDGGLSCVMGVSSGGWERLFGDEKPEYLHVFQEINGVHHAPSTP<br>GDLLFHIRAARMDLCFELASRILSDLGSSVCVVDSVQGFRYFDDRDLLGF<br>VDGTENPVAQAAVDATLIGEEDHTFSGGSYVIVQKYLHDLDKWNAIPVEQ<br>QEKIIGREKLSDIELKDADKPSYAHNVLTSIEEDGEDVDILRDNMPFGDP<br>GKGEFGTYFIGYSRKPARIERMLENMFVGNPPGNYDRILDVSRAITGTLF<br>FIPTVSFLDSVEPQPSVSQQTDDAKYIYDSPGTKGESGSLNIGSLKKEVQ<br>DE*…<br>MNNLHRELAPVSDAAWEQIEEEASRTLKRFLAARRVVDVSDPQGPA<br>FSAVGTGHVTRLEGPGDSVGAVKRQSQPVVEFRVPFILTRQAIDDVERGS<br>QDSDWSPLKEAARKIAGAEDRAVFDGYAAAGIGGIRPQSSNSPLTLPVAA<br>SGYPDVIARALDQLRVAGVNGPYHLVLGENAYTLITSGNEDGYPVLQHIH<br>RLIDGEIVWAPAIEGGVLLSTRGGDFAMDIGQDISIGYLSHTATHVELYL<br>QESFTFRTLTSEAVVSLLPSED*
KpDyP	MACPISQSVSQPVDERLTRAAIFLVVTINPGKAAEVAVRTLCGTLSSLVR<br>GVGFRILDGGLSCVMGVSSGGWERLFGDEKPEYLHVFQEINGVHHAPSTP<br>GDLLFHIRAARMDLCFELASRILSDLGSSVCVVDSVQGFRYFDDRDLLGF<br>VDGTENPVAQAAVDATLIGEEDHTFSGGSYVIVQKYLHDLDKWNAIPVEQ<br>QEKIIGREKLSDIELKDADKPSYAHNVLTSIEEDGEDVDILRDNMPFGDP<br>GKGEFGTYFIGYSRKPARIERMLENMFVGNPPGNYDRILDVSRAITGTLF<br>FIPTVSFLDSVEPQPSVSQQTDDAKYIYDSPGTKGESGSLNIGSLKKEVQ<br>DEENLYFQGGSGGHHHHHHH*
SUMO-TP_KpEnc	MSDSEVNQEAKPEVKPEVKPETHINLKVSDGSSEIFFKIKKTTPLRRLME<br>AFAKRQGKEMDSLRFLYDGIRIQADQTPEDLDMEDNDIIEAHREQIGGGG<br>SGGSGGSGGPGTKGESGSLNIGSLKKEVQDE*… MNNLHRELAPVSDAA<br>WEQIEEEASRTLKRFLAARRVVDVSDPQGPAFSAVGTGHVTRLEGPGDSV<br>GAVKRQSQPVVEFRVPFILTRQAIDDVERGSQDSDWSPLKEAARKIAGAE<br>DRAVFDGYAAAGIGGIRPQSSNSPLTLPVAASGYPDVIARALDQLRVAGV<br>NGPYHLVLGENAYTLITSGNEDGYPVLQHIHRLIDGEIVWAPAIEGGVLL<br>STRGGDFAMDIGQDISIGYLSHTATHVELYLQESFTFRTLTSEAVVSLLP<br>SED*

[1] Intergenic sequences for encapsulated constructs represented by ellipses.
[2] Stops indicated by asterisks.

Supplementary References

1. Punjani, A., Rubinstein, J.L., Fleet, D.J. & Brubaker, M.A. cryoSPARC: algorithms for rapid unsupervised cryo-EM structure determination. *Nat Methods* **14**, 290-296 (2017).
2. Goddard, T.D. et al. UCSF ChimeraX: Meeting modern challenges in visualization and analysis. *Protein Sci* **27**, 14-25 (2018).
3. Liebschner, D. et al. Macromolecular structure determination using X-rays, neutrons and electrons: recent developments in Phenix. *Acta Crystallogr D Struct Biol* **75**, 861-877 (2019).
4. Afonine, P.V. et al. New tools for the analysis and validation of cryo-EM maps and atomic models. *Acta Crystallogr D Struct Biol* **74**, 814-840 (2018).
5. Adams, P.D. et al. PHENIX: a comprehensive Python-based system for macromolecular structure solution. *Acta Crystallogr D Biol Crystallogr* **66**, 213-21 (2010).
6. Emsley, P., Lohkamp, B., Scott, W.G. & Cowtan, K. Features and development of Coot. *Acta Crystallogr D Biol Crystallogr* **66**, 486-501 (2010).
7. Emsley, P. & Cowtan, K. Coot: model-building tools for molecular graphics. *Acta Crystallogr D Biol Crystallogr* **60**, 2126-32 (2004).
8. Berka, K. et al. MOLEonline 2.0: interactive web-based analysis of biomacromolecular channels. *Nucleic Acids Res* **40**, W222-7 (2012).
9. Pravda, L. et al. MOLEonline: a web-based tool for analyzing channels, tunnels and pores (2018 update). *Nucleic Acids Res* **46**, W368-W373 (2018).
