# Supplementary material for: Structural basis for peroxidase encapsulation inside the encapsulin from the Gram-negative pathogen Klebsiella pneumoniae
Source: Nat Commun. 2024 Mar 22;15:2558. doi: 10.1038/s41467-024-46880-x (PMC10960027; doi:10.1038/s41467-024-46880-x)
Supplement: Supplementary file 3 — Description of Additional Supplementary Files [file 41467_2024_46880_MOESM3_ESM.pdf]

Description of Additional Supplementary Files

File name: Supplementary Data 1
Description: A list of enterobacterial DyP encapsulins.

File name: Supplementary Data 2
Description: Sequences used to generate the representative phylogenetic tree of enterobacterial DyP encapsulin systems.

File name: Supplementary Data 3
Description: Sequence alignment of DyP targeting peptides (TPs).

File name: Supplementary Data 4
Description: Sequence alignment of DyP encapsulin shell proteins.

File name: Supplementary Data 5
Description: ConSurf scores table.
